# Agri-environmental policies from 1960 to 2022

**DOI:** 10.1038/s43016-024-00945-8

**Published:** 2024-03-22

**Authors:** David Wuepper, Ilsabe Wiebecke, Lara Meier, Sarah Vogelsanger, Selina Bramato, Andrea Fürholz, Robert Finger

**Affiliations:** 1 Land Economics Group, University of Bonn, Bonn, Germany; 2 Agricultural Economics and Policy Group, ETH Zurich, Zurich, Switzerland

**Keywords:** Environmental economics, Climate-change policy

## Abstract

For both research and practice, it is paramount to understand what, where and when agri-environmental policies have been put in place. Here we present a database of 6,124 agri-environmental policies implemented between 1960 and 2022 in about 200 countries. The database comprises a wide range of policy types (including regulations and payment schemes) and goals (such as biodiversity conservation, safer pesticide use and reducing nutrient pollution). We illustrate the application of the database by exploring the association between economic development and agri-environmental policies and between the soil-related, agri-environmental policies of countries and their border discontinuities in cropland erosion. A strong, positive link between economic development and implemented agri-environmental policies is revealed, and it is found that 43% of all global border discontinuities in soil erosion between countries can be explained by differences in their policies.

## Abstract (web summary)

A lack of systematized information on existing agri-environmental policies poses challenges for research and practice. A new database with more than 6,000 agri-environmental policies implemented over the past six decades around the world helps fill the gap. This information enables the extraction of valuable insights for policymakers, academics and businesses.

## Main

This decade (2021–2030) has been declared by the United Nations as ‘the decade of ecosystem restoration’^1,2^. At the centre of attention is the global agricultural and food system, essential for human well-being but also responsible for a large share of greenhouse gas emissions^3^, biodiversity loss^4^, land degradation^5^ and nutrient pollution^6^.

Every year, a number of policy responses are implemented at various scales using a wide range of instruments, for example, from legislative changes to new payments for ecosystem services^2,7–11^. Yet, a consistent and coherent overview of these policies and their development over time is not available at the global level. Instead, information on different relevant policies is scattered across various sources and presented in different formats and levels of detail; thus, these policies are not directly comparable.

Here we present a comprehensive and systematic database of a wide range of policies at the intersection of agriculture and the environment, implemented not only by national entities but also by subnational and supranational entities, covering different instruments (for example, regulations, frameworks, payment programmes) and topics (for example, biodiversity, forests, fertilizers, pesticides). To create this database, we compiled and harmonized information from various existing databases and filled data gaps by adding policies found in reports, articles and government websites (see Methods for more details). We show the use of the database with two analyses: a simpler one focused on the association between agri-environmental policies and economic development, and an econometric analysis examining an agri-environmental outcome—in particular, how much the soil policies of countries have helped mitigate cropland soil erosion.

## Database overview

Our database covers roughly 200 countries and a total of 6,123 different policies for the period 1960–2022. This period corresponds to the time span of most empirical analyses in economics and political science—which, in turn, reflects the relevance of different policies to current policy-making and the availability of complementary data. Arguably, going much further back in time would have a diminishing return in terms of overall value for research; in any case, the policy database is a living resource which can be expanded as needed.

**Fig. 1:**
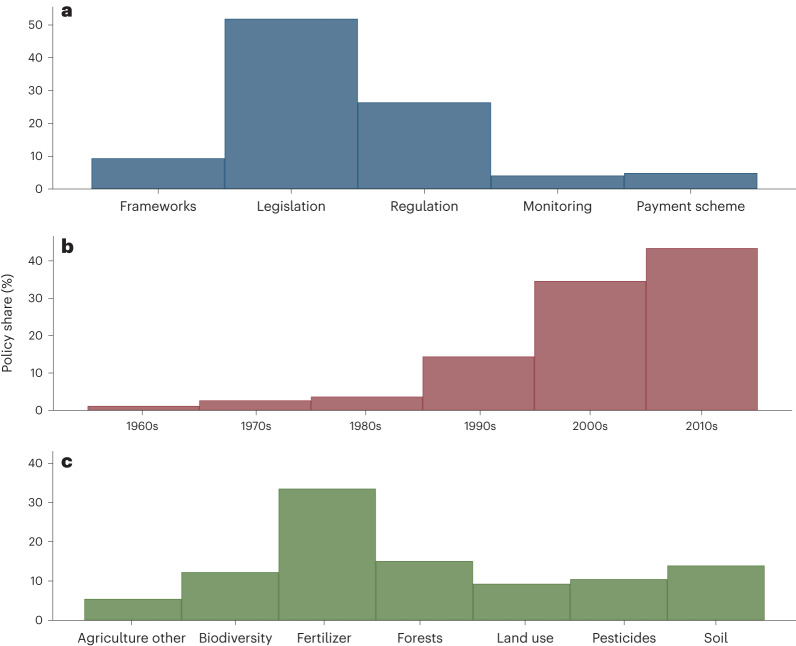
Policy characteristics (*N* = 6,124). **a**–**c**, Plots illustrate some of the ways in which the policies can be classified: policy types (**a**), timing (**b**) and targets (**c**). The keywords are not mutually exclusive. A typical policy is characterized by multiple keywords, such as ‘soil’, ‘land use’ and ‘pesticides’, for example, if a policy aims to make land use more sustainable by protecting the soil from pesticide pollution. In combination, the policy characteristics and keywords make it simple to construct policy measures. Examples could be the number of ecosystem service payment schemes that aim to conserve agricultural biodiversity per country since 2000, or fertilizer regulations since 1960. For a snapshot of the database, see Supplementary Fig. 6.

A basic summary of the dataset (including all years and countries) reveals that the most common policy type is ‘command and control’ (that is, legislations and regulations; Fig. 1a); the number of policies has steadily increased over time (Fig. 1b), perhaps reflecting their increasing priority on the political agenda; and the most common policy targets are fertilizer use and forest and biodiversity conservation (Fig. 1c). We have followed the original data sources for policy categorizations (according to which, for example, a ‘legislative change’ and a ‘regulatory change’ are not always distinguished), but we kept the original labelling as well as other complementary policy details to enable researchers to adopt their own policy categorization if necessary. For instance, this allows recategorizing or pooling together ‘legislative changes’ and ‘other regulatory changes’ together, or further dividing categories into, for example, ‘minor regulatory changes’ and ‘major regulatory changes’. Policies that could not clearly be categorized were termed ‘agriculture other’. It should be noted that for many other policies too, the boundaries between categories are fuzzy and alternative categorizations are possible. For example, depending on the application, one could combine the categories ‘biodiversity’, ‘land use’ and ‘forests’, or one could divide each of these categories into smaller subcategories, such as ‘agricultural biodiversity’, ‘grassland biodiversity’ and ‘forest biodiversity’.

As Fig. 1 illustrates, policies can be filtered according to a given set of criteria quite simply, for example, ‘legislation addressing soil conservation before 2005’ or ‘payment schemes for forest conservation implemented after 2015’. This database is, for example, useful for research in agricultural and environmental economics^7,12^, to answer questions such as how much certain policies have achieved so far, how such policies have come about^13^ or how these policies (for example, policy types, implementations and goals) have changed over time. Furthermore, these policies can be used to model the preparedness of countries for environmental change and their efforts to mitigate environmental costs, and to constraint scenarios^14,15^. Much interest also exists in understanding the kinds of policies being implemented around the world (so far, the focus has been on nitrogen pollution^11,16^ and climate change^17,18^) and to compare environmental policies across regions, countries and continents.

## Global distribution of agri-environmental policies

**Fig. 2:**
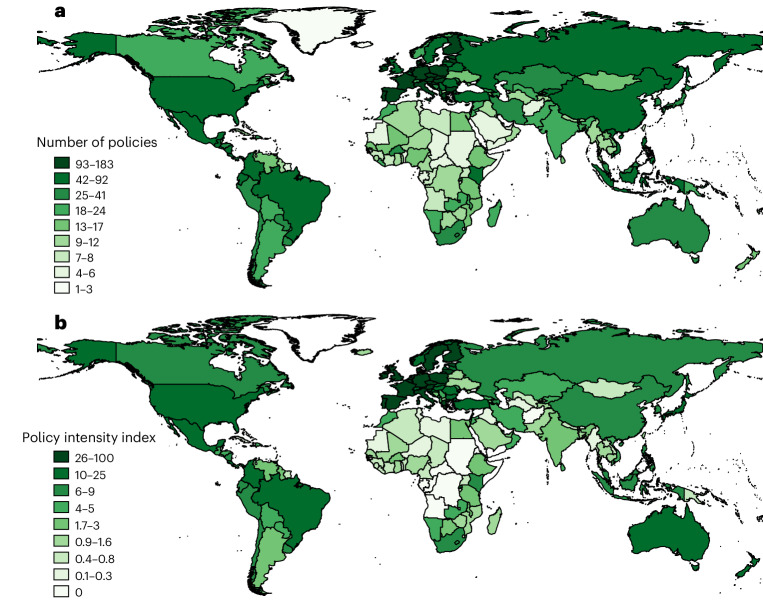
Number of agri-environmental policies and policy intensity index per country. **a**, A simple count of each country’s regulations, frameworks, payment programs and so on may offer hints into agri-environmental policy efforts, but ignores important policy characteristics such as ambition, targeting, stringency and enforcement. **b**, An alternative is to use a more complex metric by taking into account the general policy stringency and enforcement of these countries or the level of corruption they face. Arguably, agri-environmental policies are on average more effective when implemented by and in countries that generally have more stringent and better-enforced environmental policies and lower levels of corruption. By weighting the simple measure shown in **a** by these factors, an augmented measure can be obtained, as shown in **b** (Methods). EU member states have their own policies plus those of the EU.

Agri-environmental policies are unequally distributed across countries globally (Fig. 2). European countries have the most policies, which is to a large degree driven by the existence of the European Union (EU). Comparatively, for example, African countries have implemented the least public agri-environmental policies over the same period.

Figure 2a shows a simple count of policies, which is not a precise measure of actual policy intensity or policy effectiveness, but a good starting point. Clearly, one ambitious, well-designed policy (for example, stringent, well enforced and precisely targeted) might be more effective than multiple less ambitious policies. To improve the policy measure, the policies of countries can, for example, be weighted according to their policy stringency and enforcement^19^, and corruption levels^20^, as shown in Fig. 2b. The assumption for such an augmented approach is that these country-level weights positively correlate with, and thus capture, policy design and implementation, available budget, institutional capacity and so on. Generally, the patterns in Fig. 2a,b look similar (correlation coefficient, 0.86) and it is an empirical question whether the difference is practically relevant for an analysis. It is worth noting, however, that between-country differences are magnified under the augmented approach because there is a generally positive relationship between the number of environmental policies and the policy stringency, enforcement and corruption control of countries. For some countries (for example, Russia and China), the augmented approach lowers the measure compared with the mere count of their policies, whereas for other countries (for example, Canada and Australia), the measure is raised.

Regularly, policy design questions will be of first-order interest in analyses involving our database^21–23^. For example, even within the narrow domain of a specific policy type and goal, different countries and different regions within countries may have designed a given policy in distinct ways, with implications for these policies’ effectiveness and efficiency^24,25^. Our database can aid such an investigation at the global level and for a long period of time—for instance, one can match it with information on policy designs and mixes to examine how contextual factors and policy designs interact; when and how policy change has come about; when and where goals and means have changed; whether minor design changes over time have generated change compared with punctuated, ‘transformational’ change; and many more.

## Case study 1

There is an extensive literature on the relationship between economic development and environmental outcomes^26^. On the one hand, rising incomes lead to increased consumption, with associated negative environmental impacts. On the other hand, political priorities might change in a pro-environmental direction, and higher-income countries might implement more environmental policies. Using the database, we can look at this question empirically by examining this relationship at global level and for policies at the intersection of agriculture and the environment.

**Fig. 3:**
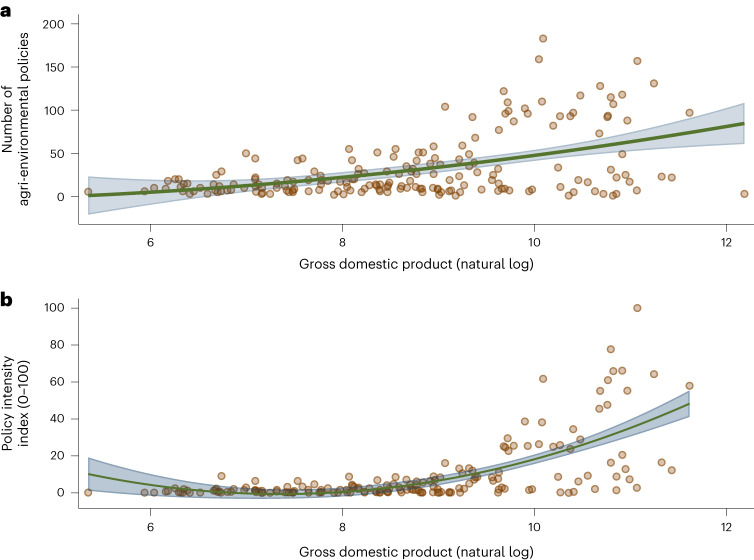
Relationship between GDP and number of agri-environmental policies. **a**, Each dot represents a country in the database. The fitted line and corresponding 95% confidence interval to indicate the general trend come from a linear regression. Countries with a higher GDP implement more agri-environmental policies than those with a lower GDP (*N* = 186). **b**, As in Fig. 1, we can use an augmented policy effort measure (‘policy intensity index’) that weights the simple number of policies by the average policy stringency and enforcement of countries as well their level of corruption. The empirical relationship between this measure and the GDP of countries is even stronger than the relationship with the simpler measure shown in **a**. Whereas the relationship with the simpler measure is approximately linear, the relationship with the augmented measure is approximately exponential. The fitted line and corresponding 95% confidence interval to indicate the general trend come from a nonlinear regression (*N* = 176). As shown in Supplementary Fig. 5, a similar but reversed pattern appears if one replaces the GDP of countries with the agricultural share of their GDP.

By regressing the number of agri-environmental policies of countries on their gross domestic product (GDP), we see a basically linear, positive relationship—but also an increasing variance, as a growing concern for the environment does not develop at the same pace and extent in every country. Several high-income countries, such as Kuwait, Qatar and the United Arab Emirates, have so far implemented a similarly low number of agri-environmental policies as countries with much lower GDP (Fig. 3a). If we turn again to the augmented policy index introduced earlier, the relationship turns from linear to exponential given a positive interaction effect between the number of agri-environmental policies and beneficial contextual factors, such as high stringency and enforcement of policies and comparably low corruption (Fig. 3b). This is especially pronounced among EU countries.

## Case study 2

For our second case study, we revisit the study of Wuepper et al.^27^ on soil erosion discontinuities at country borders, which uses a spatial regression discontinuity design to separate the share of soil erosion that is explained by country-level variables and the share of soil erosion that is explained by environmental and local variables. The study finds that almost half of the global rate of soil erosion is shifted by socio-economic country characteristics, such as national institutions, policies, markets and culture. However, for a lack of comprehensive policy data, finer mechanisms could not be identified beyond suggestive evidence about the importance of agriculture^27^.

**Fig. 4:**
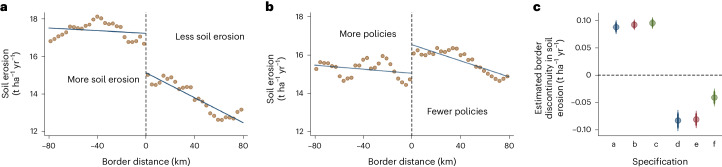
Global border discontinuities in soil erosion. **a**, Spatial distribution of cropland soil erosion rates (t ha^−1^ yr^−1^) within 80 km of international borders. The brown dots are 2 km long, local averages; the blue lines are fitted spatial trends. The dotted line is the average global border. All data points from countries with a higher erosion rate than their pairwise neighbour are plotted to the left of the border, whereas all data points from countries with a lower erosion rate than their pairwise neighbour are plotted to the right. A country effect is indicated by the discontinuity right at the border. **b**, Countries sorted according to their number of implemented soil policies. All data points from countries with more soil policies than their pairwise neighbour are plotted to the left of the border, whereas all data points from countries with fewer soil policies than their pairwise neighbour are plotted to the right. A policy effect is indicated by the discontinuity right at the border. **c**, Estimated coefficients (the centres of the bars) and corresponding 99%, 95% and 90% confidence intervals (the bars) from a spatial regression discontinuity design. The first three coefficients show the percentage soil erosion change that is estimated for a grid cell being in the country that causes more soil erosion compared with one being located in the neighbouring country that causes less (a–c). The last three coefficients show the percentage soil erosion change that is estimated for a grid cell being in the country that has implemented more soil policies compared with one being located in the neighbouring country that implemented fewer (d–f). (*N* = 15,687,325).

Here we examine the extent to which differences in the soil-related policies of countries—as extracted from our database—explain the large border discontinuities in soil erosion between countries. We first replicate the identification of general border discontinuities in soil erosion on cropland from Wuepper et al.^27^, sorting countries pairwise at international borders according to whether they have higher or lower rates of soil erosion (Fig. 4a, and a–c of Fig. 4c) and then by whether they have implemented more soil policies, again pairwise, comparing neighbouring countries at each shared border (Fig. 4b, and d–f of Fig. 4c). For both country and policy discontinuities, we show three specifications: a baseline (a and d of Fig. 4c), one controlling for natural erosion determinants such as rainfall and soil characteristics (b and e of Fig. 4c) and one controlling for the socio-economic characteristics of countries (GDP, Environmental Performance Index (EPI), corruption and private property index) (c and f of Fig. 4c). Comparing estimated coefficients ‘a, b, c’ with ‘d, e, f’, we find that at least 43% of the country effect is explainable by the policies of countries.

## Conclusion

Both analyses—of the relationship between economic development and agri-environmental policies, and of the role of public policies in the soil erosion effect of countries—add important insights to the existing literature. An early study by Grossman and Krueger^28^ showed that environmental indicators (on air pollution and river water quality) first deteriorate with economic development but then improve again. However, as discussed by Jayachandran^26^, based on subsequent empirical research on the topic, the relationship between these variables is complex and context dependent. This means that it is advantageous to ask narrow and well-defined questions, such as what the relationship is between economic development and the implementation of agri-environmental policies. The database presented here provides the necessary data to look exactly at this question.

The role of public policies in explaining between-country differences in environmental performance is an equally important question. There are empirical estimates for some environmental outcomes, notably climate change mitigation^29^, pesticide pollution^30^ and forest conservation^31^. In each case, public policies are found to improve environmental outcomes, but the effect is small for overall climate change mitigation (in the overall economy) and comparatively larger for pesticide pollution and forest conservation. Our database includes soil-focused policies, so we can estimate their impact on global soil erosion, another major agricultural sustainability issue^15^—and this reveals a large effect (Fig. 4): about 43% of the overall impact of countries is explained by their policies.

Similar previous work to compile data on the agri-environmental policies of countries must be acknowledged, which is the basis of this current effort. For instance, Kanter et al.^11^ collated a global database of nitrogen policies, Ezzine-de-Blas et al.^32^ provided a global database on payment for ecosystem services, Eskander and Fankhauser^29^ examined legislative changes to address climate change, Tang et al.^33^ provided a global database on pesticide regulations and Börner et al.^8^ offered information on forest conservation policies. Moreover, Olczak et al.^18^ created a global dataset on methane policies and Yang et al.^16^ have focused specifically on nitrogen policies in Southeast Asia. There also exist multiple online databases with inputs to the studies above and this study^34–37^ (Methods). Still, our database can be conveniently used—either directly (for example, to check whether a political entity has implemented certain kinds of policies at some point in time) or in combination with other data sources (for example, merged at the national level via International Organization for Standardization (ISO) country codes).

## Methods

The policy database presented here is fully modular and can be expanded and reused in many forms and directions. Currently, it includes the following general themes: agricultural and related land use, nitrogen fertilizer, pesticides, biodiversity, forests and soils. We do not make any distinction between policies, laws and legislations, and treat all of them as policies, acknowledging that in some disciplines and contexts, there is an important distinction.

To be included in the database, a policy had to fulfil a number of conditions. First, it must be relevant for agriculture. This does not mean it must be strictly an agricultural policy (for example, a general groundwater policy can be highly relevant for agriculture), but many environmental policies that do not have a direct link to agriculture (for example, policies focused on the protection of marine biodiversity or industrial pollutants) were excluded. Second, it must be a ‘public policy’ (here understood as a policy that is mostly implemented by non-governmental actors, such as an industry or even single companies, or large international organizations such as the World Bank). We have included policies that are supported by an international or private organization (for example, REDD+ (Reducing Emissions from Deforestation and Forest Degradation), World Bank) as well as policies from the EU as it consists of governmental actors and is integrated into national and subnational policy-making albeit being supranational. In the database, EU policies are separately reported from the policies of individual countries, but for most applications, these should be added to the policies of EU member countries.

The database is not exhaustive but aims to cover the most relevant policies. In an effort to capture as many relevant policies as possible, we have conducted cross-checks (that is, a researcher who collected policies on some countries double checked other countries) and discussions with experts on specific countries. Some policies were probably left out (including those that are not published digitally or in a language that is not using a Latin alphabet); however it is to be expected that more influential and important policies are also easier to identify.

The policies in our database include both ‘command and control’ and ‘incentives based’ policies (that is, they include policies that imply that landowners are mandated to change their behaviour and those that imply that landowners are offered payments to do so). Here we describe from where and how each of these two types were derived. The final database contains country names and ISO3 codes for identification, two columns of keywords (for example, ‘agriculture’ and ‘soil erosion’), the policy type, the year of policy implementation and up to three reforms, whether a policy was discontinued, additional descriptors (more detailed than the keywords and unique for each policy), the title of the policy, a web link, a pdf with the policy text and an abstract for 6,124 agri-environmental policies.

### Data records

Three datasets were used. The main dataset contains all policies at the intersection of agriculture and the environment, implemented between 1960 and 2022, with full details. The baseline version is published on Zenodo (Data Availability) and will be updated and augmented regularly. It currently contains 6,124 agri-environmental policies implemented at different scales.

The second dataset contains only national policies, merged with multiple other country-level variables, capturing economic, institutional and various other characteristics, collapsed across all years. We use the second dataset to create the maps in Fig. 2 and Supplementary Figs. 1–4, as well as the plots shown in Fig. 3 and Supplementary Fig. 5.

The third dataset contains global high-resolution data on cropland soil erosion rates at a resolution of 500 × 500 m, matched with our policy data and selected control variables. Supplementary Table 1 shows the variables in the main dataset (POLICY DATABASE 1.0). Supplementary Table 2 shows the variables in our national-level dataset (COUNTRY DATA 1.0). Supplementary Table 3 shows the variables in our soil erosion dataset (SOIL POLICIES 1.0).

### Command-and-control policies

Three major databases exist from which command-and-control policies can be filtered and curated: ECOLEX (https://www.ecolex.org/), FAOLEX (https://www.fao.org/faolex/en/) and SOILEX (https://www.fao.org/soils-portal/soilex/en/). ECOLEX has already been used to quantify the nitrogen fertilizer^38^ and climate change policies^29^ of countries. FAOLEX and SOILEX add more details on agriculture-specific and soil-specific topics. Together, these three databases are the web’s most comprehensive and reliable source of agri-environmental policies. They are run by the Food and Agriculture Organization (of the United Nations), International Union for Conservation of Nature and United Nations Environment Programme and contain information on national, international and regional laws, categorized by sector, issue, policy type and many more. Information that is not available from these databases includes costs and budgets, enforcement and implementation on the ground, and quantitative measures more generally, for example, a measurement of ambition or effectiveness. Most information is qualitative, but in such a detail that simple quantitative measures can be derived.

To find the relevant policies for our research, we used various search terms. The starting points were the broader topics that we aimed to include, such as land use or forests (Fig. 1). We then searched for alternative keywords, first by searching for related terms and then by searching for terms that came up during the search. For example, for agricultural land use, we searched for general terms like ‘agricultural land’ and even generally ‘land use’, and specific terms, such as ‘pastoralism’ or ‘cropland’. For forest policies, keywords included ‘forestry’, ‘forest protection measures’, ‘agro-forestry’, ‘timber’, ‘logging’, ‘extraction’ and other similar terms. Also, the keyword ‘biodiversity’ produces many forest policy results, but also separate non-forest biodiversity policies. The databases also provide an object filter, and keywords include ‘forestry’, ‘agricultural and rural development’, ‘land and soil’, and ‘wild species and ecosystems’. Only legislations were considered and not, for example, bilateral treaties, and only the main policies were retained, based on two criteria: scope (for example, the main focus is one of our subjects, for example, ‘forests’ or ‘soils’, and in relation to agriculture, so not, for example, soil contamination from mining) and scale (a major legislative change with potentially important implications, not, for example, a small local policy change). We recorded policy reforms in addition to the initial introduction of a policy but did not count these as new policies. Operationally, this means many policies have more than one date, with each date showing one reform, and sometimes a note that the policy was discontinued.

### Incentive-based policies

For incentive-based policies, Ezzine-de-Blas et al.^32^ created a quantitative database of 55 payment-for-ecosystem service schemes around the world. Moreover, Chabé-Ferret and Voia^39^ and Chabé-Ferret and Voia^40^ collected data on payment schemes for forests and grasslands. An overview of REDD+ projects can be found on the IDRECCO database^34^. In addition, we searched for relevant policies using Google as well as published articles and reports. The majority of the relevant incentive-based policies offer either payments for the adoption of more sustainable agricultural practices to farmers (often in higher-income countries) or payments for forest conservation to landowners (often in lower-income countries), but others also exist (for example, payments to farmers for measured biodiversity outcomes). Most of these policies were found using the terms ‘payments for ecosystem services’, ‘agri-environment scheme’ and variations thereof.

An important additional inclusion criterion for incentive-based policies is whether or not they are mostly public and governmental. By contrast to command-and-control policies, incentive-based policies are implemented by a wide range and combination of actors, including companies, international supernational organizations and programmes, and so on. The database includes only policies that are mainly controlled by a regional or national government. For example, we included only REDD+ initiatives that were mainly financed by the host country and not otherwise (assuming that the actor who finances the initiative is the most powerful actor).

Here, too, another inclusion criterion is the importance and size of the policy. This is even more relevant for incentive-based policies because many schemes start as small pilots or are intended to be only locally implemented. Incentive-based policies that did not cover at least one entire subnational administrative unit (for example, federal state, region, canton) were thus excluded, especially policies implemented at the level of watersheds—which may even combine local and private actors.

### Technical validation

During data collation, the entered data were constantly cross-checked, that is, one researcher entered data and another researcher confirmed correctness. The final product was then finally checked once more for duplicates and errors, going through the entire list of 6,124 policies once more.

### Data combination and augmentation

All policies are collated in a single database that can be filtered by various keywords and matched with additional information. The main variable to match additional national variables is the country ISO3 code, which allows simpler matching than matching using country names that might slightly differ between datasets. A potential main use of this policy database is for quantifying regional or national agri-environmental policy efforts. For example, the research question might be how much public agri-environmental policies have reduced the risk of pesticide or fertilizer pollution, or how much such policies have protected forests, or how much soil erosion could be avoided by implemented public policies. One way to analyse these questions is to adopt an econometric research design such as a regression discontinuity design^41^ or a difference in differences design^42^, for example. That way, one can estimate whether there is a discontinuity at an administrative boundary (for example, an international border) between political entities that have implemented a relevant policy and those that have not—possibly even over time, showing that there was no discontinuity before and then one arose after the policy was implemented^43^, or how temporal dynamics have changed over time between those political entities that implemented a relevant policy and those that have not.

### Policy intensity indices

Sometimes, it can be useful—or even necessary—to summarize more than one policy per political unit, in a sort of ‘policy intensity index’. For example, Eskander and Fankhauser^29^ recently asked the question how much greenhouse gas emissions (if any) were reduced by national climate legislation changes. Over time, many countries implemented many changes, so Eskander and Fankhauser^29^ chose the cumulative number of climate change mitigation laws as their explanatory variable (in their main specification divided into more recent and older laws). The main assumption here is that the number of policies and changes correlates sufficiently closely with the actual policy ‘effort’ or ‘intensity’. Knill et al.^44^ and Schaffrin et al.^17^ use six indicators to measure this—namely, objectives, scope, integration, budget, implementation and monitoring—for a small selection of countries. Their work aimed to measure how policy intensity changes over time. For example, the same policy might become more effective over time if its budget is increased or monitoring is improved. Zhang et al.^45^ recently proposed a machine learning approach that aims to collate a wide range of policy indicators and produces a policy intensity indicator for China between 1978 and 2019.

Here we consider various relatively simple measures of policy intensity. We start by mapping the number of national agri-environmental policies per country since roughly the 1960s. We then adjust this measure by weighting the number of policies by the Environmental Policy Stringency Index and Bayesian Corruption Index^20^ of countries, just to illustrate how this can be done. This augmentation possibly improves the simpler measure of counting policies. We show maps, both here and in the Supplementary Information, that demonstrate how much the different measures differ from each other, for example, in terms of relative country rankings. In the Supplementary Information, we show a strikingly strong correspondence between our different policy intensity measures and the 2022 EPI^46,47^. The database is open access and allows for constant updating and augmentation by any user. Moreover, the collection of all variables has been done manually, but our intention is to have it automated to some extent^48^.

#### Policy stringency and enforcement

A measure of the environmental policy stringency and enforcement in countries has been developed by the World Economic Forum. This is based on a survey of business leaders^19^. The respondents are asked the following two questions:

1. How would you assess the stringency of your country’s environmental policy? (Scale 1 = very lax; 7 = among the world’s most stringent)
2. How would you assess the enforcement of environmental regulations in your country? (Scale 1 = very lax; 7 = among the world’s most rigorous)

These measurements are based on those developed by Dasgupta et al.^49^, which have subsequently been augmented and expanded by Eliste and Fredriksson^50^, and Wagner and Timmins^51^. See also Dechezleprêtre and Sato^52^ and Sauter^53^ for reviews. These two variables have obvious limitations (they are not policy specific, only business leaders have been surveyed and they are general and simple overall) but do contain helpful information—and currently, there is not a superior alternative at the global level.

#### EPI

Based on 40 different performance indicators across 11 categories of issues, the EPI^46,47^ evaluates countries regarding their climate change performance, environmental health and ecosystem vitality. In contrast to the above-discussed survey-based indicators, the EPI is purely data driven and based on objective measurements. The 11 indicator categories are (1) climate change, (2) air quality, (3) sanitation and drinking water, (4) heavy metals, (5) waste management, (6) biodiversity and habitat, (7) ecosystem services, (8) fisheries, (9) acid rain, (10) agriculture and (11) water. The EPI combines multiple variables of completely different categories, including policy outputs and policy outcomes, which should be noted when this variable is used.

#### Corruption

The level of corruption each country faces can influence agri-environmental policies in multiple important ways. Potentially, corruption can mean that policies are not designed to be effective^8,54^ or that they are not implemented in a way that makes them effective^55,56^. There are alternative international corruption measures available, such as the Corruption Perception Index of Transparency International^57^ and the Worldwide Governance Indicators of the World Bank^58^. Arguably the most reliable measure currently available is the Bayesian Corruption Index of Standaert^20^. It is common knowledge that corruption is difficult to measure because it is usually hidden. Thus, as for the policy stringency and enforcement indicators above, also corruption in countries is measured using subjective perceptions. The Bayesian Corruption Index is an amalgamation of the corruption perceptions of each country’s inhabitants, companies operating there, non-governmental organizations and officials working both in governmental and supragovernmental organizations. It then combines the information of 20 different surveys and more than 80 different survey questions that cover the perceived level of corruption. Also, this variable has its clear limitations, in particular the fact that it is based on perceptions that cannot be independently verified.

### Public policies and soil erosion

It has been estimated that at national borders, the global rate of soil erosion changes (on global average) by about 1.4 t ha^−1^ yr^−1^ (ref. ^27^). This cannot be explained by natural causes but is caused by socio-economic differences between countries^27^. So far, however, it remained unclear where the impact of countries comes from exactly^27^. Potential explanations are many, including differences in institutions, policies, economic structures and development, culture, trade and many more. Wuepper et al.^27^ could only identify that the impacts of countries are associated with agricultural differences between the countries. To estimate how much of the impact of countries is explained by differences in their public policies, we use a spatial regression discontinuity design, like in the original study^59^. The first step of the approach is to focus only on border areas around the world, where many confounding factors are relatively similar on either side (for example, close to a typical national border, environmental determinants of soil erosion—such as topography, soil types and rainfall—tend to be similar, whereas they tend to be quite different when comparing countries to each other as a whole). This situation resembles a naturally occurring experiment, in which a grid cell of land is assigned to the policies of the country to which it belongs, which might be the country with more policies (*D* = 1) or with less (*D* = 0), and we control for the distance from each grid cell to the border, which controls for spatially continuous confounding variables (for example, remaining differences in topography, rainfall, soil types). We estimate the regression discontinuity design in two versions. First, we estimate the average soil erosion border discontinuity between countries with a higher rate (*D* = 1) and those with a lower rate (*D* = 0), to identify the overall border discontinuity between countries. Second, we estimate the average soil erosion border discontinuity between countries that implemented more soil policies (*D* = 1) and those that implemented less (*D* = 0), for a comparison of magnitudes. The ratio of the second to the first estimate approximately quantifies the role of the public policies of countries in their overall impact^27,59^. This can be expressed as:

1 $${Y}_{i}=\alpha +\tau {D}+{\beta }_{1}{{X}_{i}}^{\mathrm{A}}+{\beta }_{2}{{X}_{i}}^{\mathrm{B}}+{\bf{\uptheta }}+{\varepsilon }_{i}$$

with *Y*_*i*_ being the rate of soil erosion on cropland, which varies at the grid-cell level with a resolution of 500 m × 500 m; *α* is a constant; and *D* is the treatment variable; we control for the border distance of each grid cell (*X*_*i*_) separately on either side of each border (side A and side B), and geographic fixed effects **θ**, and *ε*_*i*_ is the error term.

### Reporting summary

Further information on research design is available in the Nature Portfolio Reporting Summary linked to this article.

### Supplementary information

Supplementary Information Supplementary Figs. 1–6 and Tables 1–3.

Reporting Summary

## Data Availability

All data are available via Zenodo at 10.5281/zenodo.10422463 (ref. ^60^).

## Code availability

All code is available via Zenodo at 10.5281/zenodo.10422463 (ref. ^60^).
