# Supplementary material for: Agri-environmental policies from 1960 to 2022
Source: Nat Food. 2024 Mar 22;5(4):323–31. doi: 10.1038/s43016-024-00945-8 (PMC11045445; doi:10.1038/s43016-024-00945-8)
Supplement: Supplementary file 1 — Supplementary Figs. 1–6 and Tables 1–3. [file 43016_2024_945_MOESM1_ESM.pdf]

nature food

Analysis https://doi.org/10.1038/s43016-024-00945-8

Agri-environmental policies from 1960 to 2022

In the format provided by the authors and unedited

Contents

Supplementary Fig. 1. Number of National Policies per Country without EU Policies

Supplementary Fig. 2. Stringency and Enforcement of Agri-Environmental Policies

Supplementary Fig. 3. Policy Intensity Index Corruption Weighted

Supplementary Fig. 4. Environmental Performance Index

Supplementary Fig. 5. The Relationship Between Agricultural GDP Share and Number of Agri-Environmental Policies

Supplementary Fig. 6. Illustrative Snapshot from the Database

Supplementary Table 1. Main Dataset Variable Overview

Supplementary Table 2. Variables in the Country-Level Dataset

Supplementary Table 3. Variables for the Soil Erosion Analysis

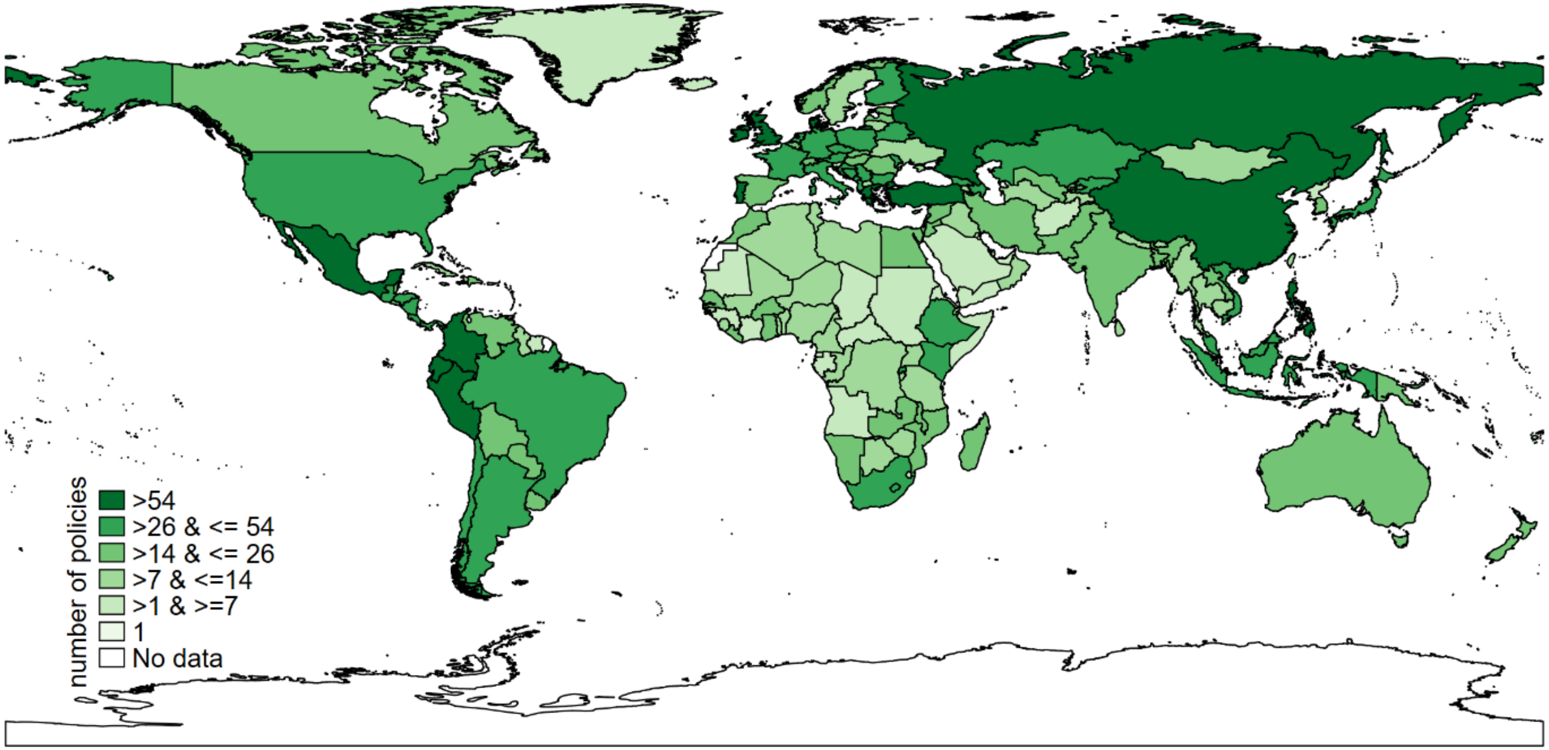

Supplementary Fig. 1. Number of National Policies without European Union Policies (N = 4542). This map shows the number of national agri-environmental policies per country without those of the European Union. It can be seen that a large share of the agri-environmental policies of the EU member countries are EU policies. For most analyses, the EU policies should thus be added to the national policies of the EU member countries (shown in the main text).

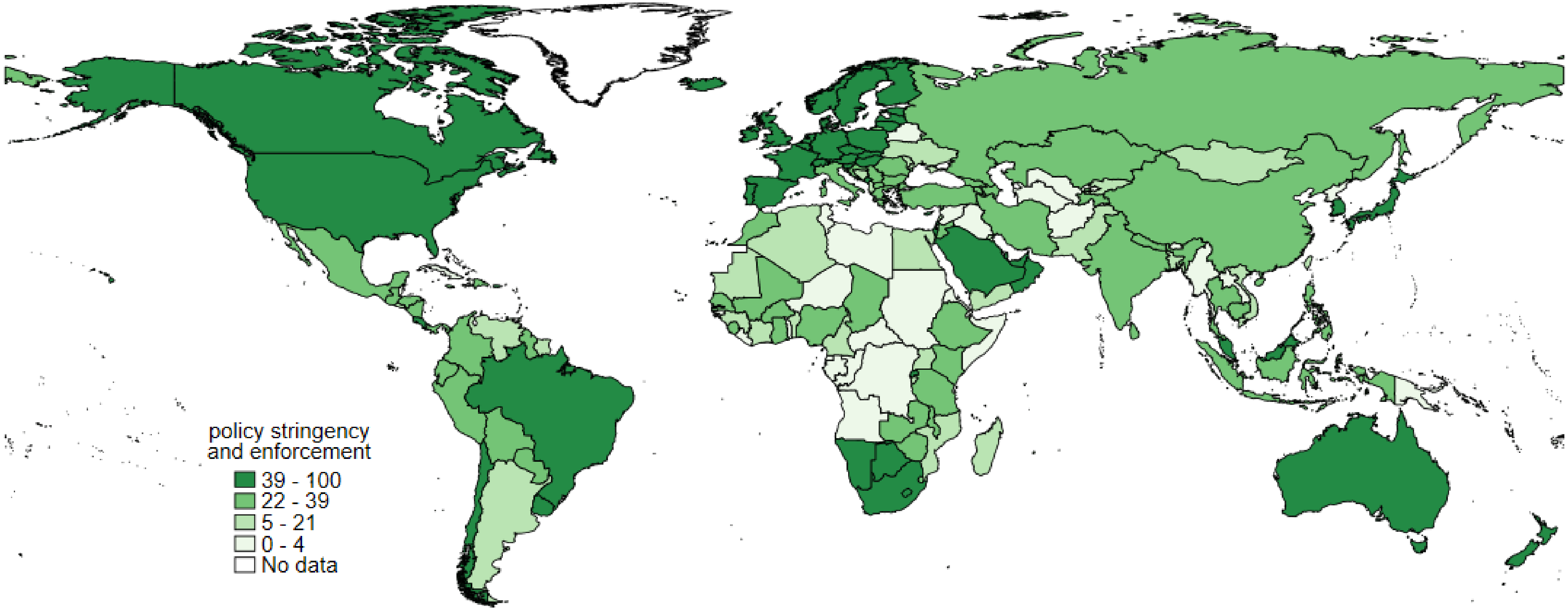

Supplementary Fig. 2. Stringency and Enforcement of Agri-Environmental Policies (imputed) (N = 186)[19] The stringency and enforcement of environmental policies is a crucial policy design feature. The same number of policies will have strikingly different effects depending on whether they are stringent and strictly enforced, or not. This map shows which countries globally are rated to implement stringent and well-enforced environmental policies (each given an equal weight here).

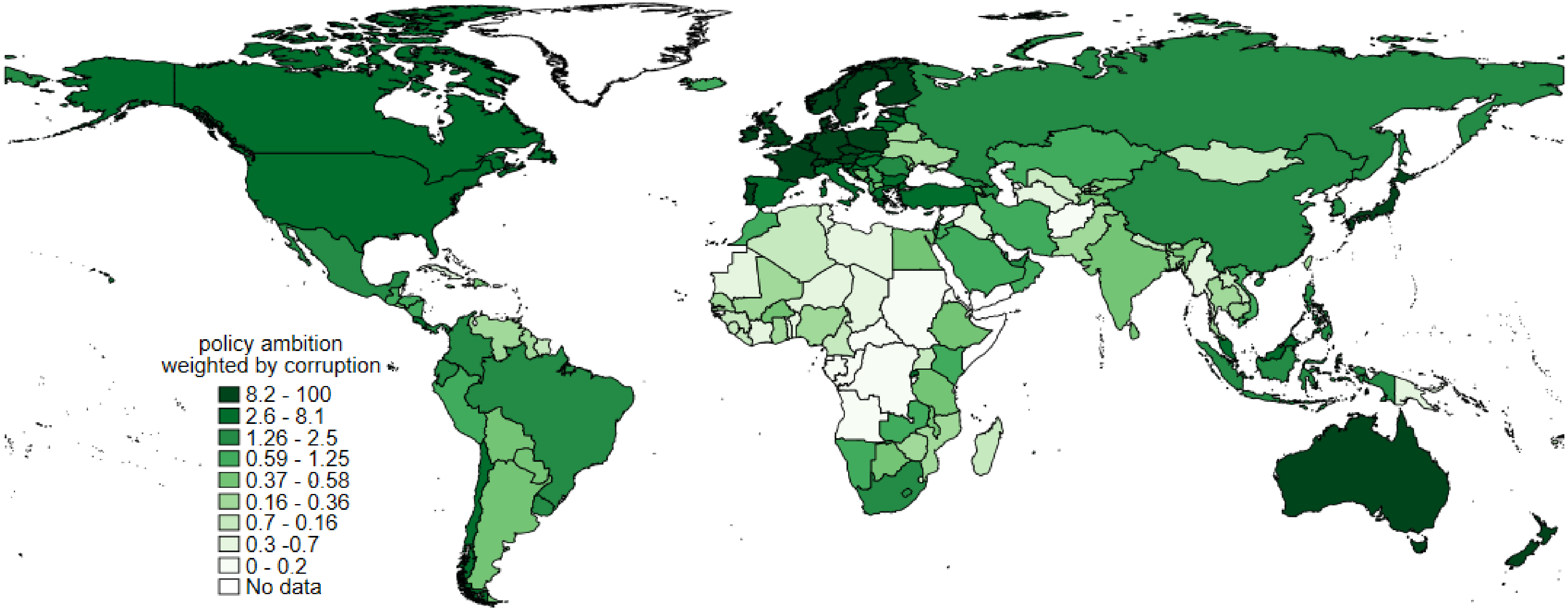

Supplementary Fig. 3. Policy Intensity Index Corruption Weighted (imputed) (N = 180). Corruption is a contextual factor that can make agri-environmental policies less effective and efficient via multiple pathways, such as negatively affecting targeting, stringency, monitoring, and enforcement of policies. This map shows the number of agri-environmental policies per country (**Fig. 2** in the main text), only weighted by countries' Bayesian corruption index.

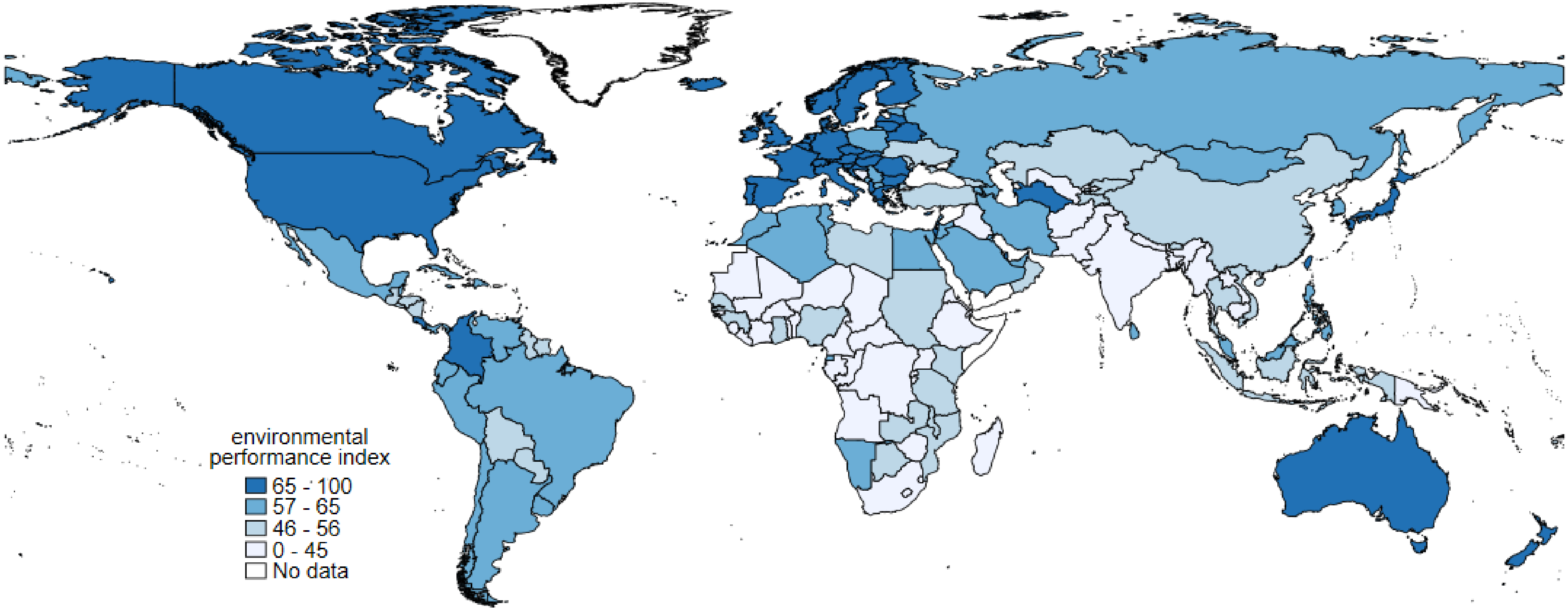

Supplementary Fig. 4. Environmental Performance Index (N = 186)[47]. A visual comparison between the above presented agri-environmental policy maps (Fig. 2 and 3 in main text, Figure S3 in the Supplementary Materials) and a Map of the Environmental Performance Index shows strong correspondence

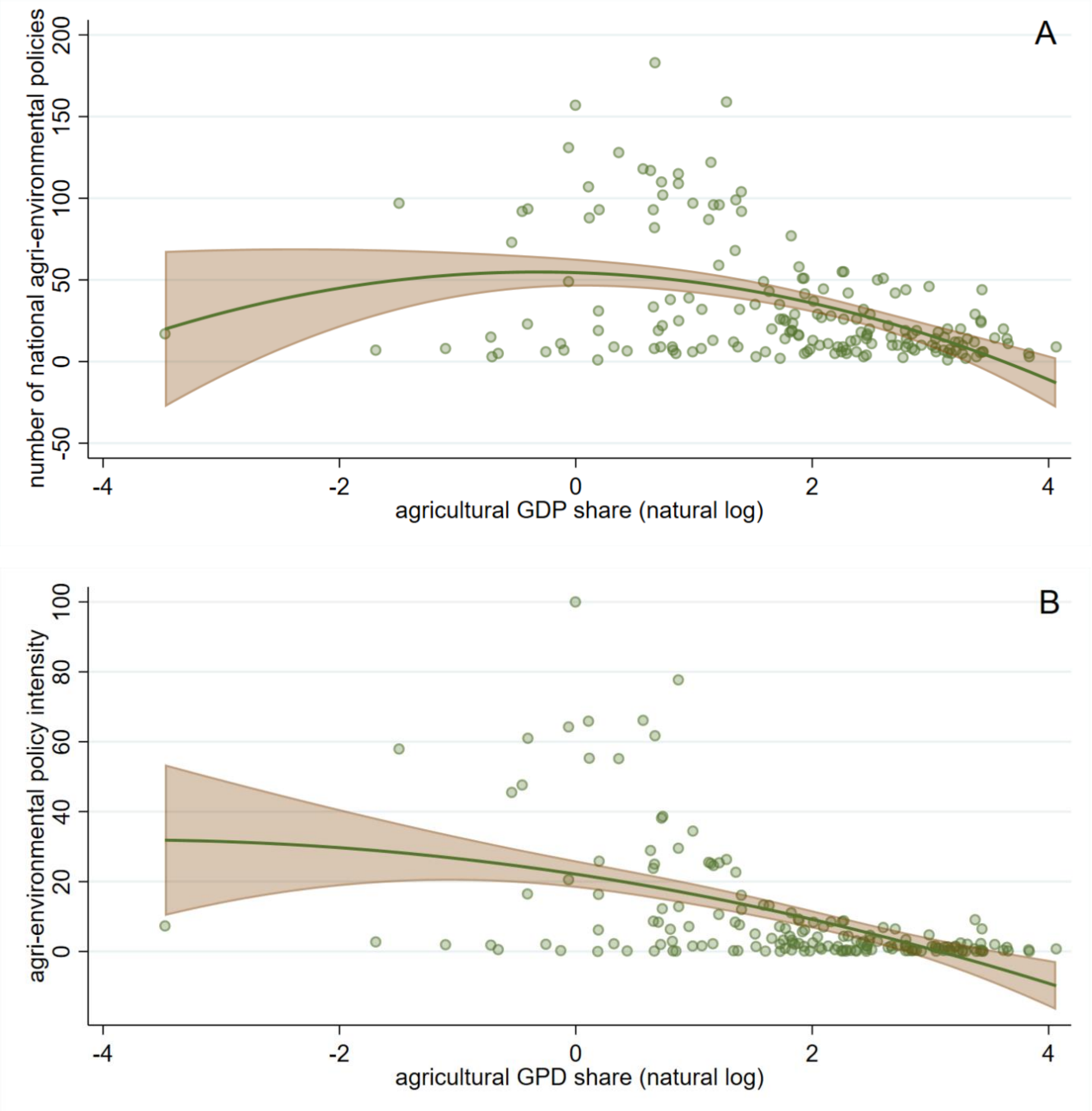

Supplementary Fig. 5. The Relationship Between Agricultural GDP Share and Number of Agri-Environmental Policies. In both panels A and B, each dot represents a country in the database. The fitted line and the 95% confidence interval around it are produced by a non-linear regression.

	ISO3	Country	Keyword1	Keyword2	PolicyType	Scale	Year1	Year2	Year3	Initiated	Descriptors	Title	Link	RelatedFile	Abstract
412	AUT	Austria	Fertilizer		Regulation	Regional	1996			1996	groundwater; water sup	Ordinance by the	LEX-FAOC(	aut7918.pdf	An Ordinance to regulate the
413	AUT	Austria	Fertilizer		Regulation	National	2010			1991	potable water; MRL-max	Quality Target Orc	LEX-FAOC(	Quality Target	The Ordinance contains at the
414	AUT	Austria	Fertilizer		Monitoring	National	2002			2002	air quality/air pollution;	Emission Cadastr	LEX-FAOC(	aut90976.pdf	The present Ordinance lays dc
415	AUT	Austria	Fertilizer		Regulation	National	1983			1983	pollution control; air qua	Agreement on est	LEX-FAOC(	Agreement on	An Agreement between the fe
416	AUT	Austria	Fertilizer	agriculture	Regulation	National	2014			2004	fertilizers/nutrients; pac	Fertilizers Ordinai	LEX-FAOC(	Multiple files -	The present Ordinance lists in
417	AUT	Austria	Fertilizer	agriculture	Regulation	Regional	2013			1985	fertilizers/nutrients; soil	Sewage Sludge La	LEX-FAOC(	aut92434.pdf	The present Law lays down pr
418	AUT	Austria	Pesticide	Agriculture	Legislation(re	National	1990			1990	basic legislation; plant pi	Pesticides Act.	LEX-FAOC(	aut3412.pdf	The present Act sets out detai
419	AUT	Austria	Forest		Payment Sche	National	2019			2019			https://fo		ForestValue ERA-NET Cofund
420	AUT	Austria	Agricultur		Legislation	National	1971			1967	agricultural developmen	Basic Agricultural	LEX-FAOC:	aut131048.pdf	Article 1 of the above-mentior
421	AUT	Austria	Fertilizer		Regulation	Regional	2016			2000	basic legislation; air qual	Burgenland Air Pc	LEX-FAOC:	aut111677.pdf	The purpose of the present La
422	AUT	Austria	Agricultur		Payment Sche	National	2000	2007	2015	2000			https://wv	https://info.br	
423	AUT	Austria	Fertilizer		Regulation	Regional	2015			2010	air quality/air pollution;	Heating System O	LEX-FAOC:	aut167986.pdf	The present Ordinance impler
424	AUT	Austria	Agricultur		Legislation	National	2015			2001	fertilizers/nutrients; pes	Varieties Protectic	LEX-FAOC(	aut89353.pdf	Article 8 of the Agricultural An
425	AUT	Austria	Fertilizer		Regulation	National	1990			1983	basic legislation; instituti	Environment Fun	LEX-FAOC(	Environment F	The present Act lays down prc
426	AUT	Austria	Soil		Legislation	National	2015			1994	basic legislation; fertilize	Fertilizers Act	LEX-FAOC(	aut89394.pdf	An Act to regulate the trade in
427	AUT	Austria	Fertilizer		Monitoring	National	2007			2007	emissions; data collectio	Emission Stateme	LEX-FAOC(	Emission State	The present Ordinance lays dc
428	AUT	Austria	Forest		Payment Sche	National	2016			2016			https://wv		Forest for Water
429	AUT	Austria	Fertilizer		Regulation	National	2001			2001	waste disposal; waste m	Compost Ordinan	LEX-FAOC(	aut90980.pdf	The present Ordinance lays dc
430	AUT	Austria	Fertilizer		Regulation	National	2013			1997	thermal and medicinal w	Salzburg Thermal	LEX-FAOC(	aut91643.pdf	The present Law lays down pr

Supplementary Fig. 6. A Snapshot from the Database. Shown here are a few of the policies recorded for Austria.

Variable	Explanation
ISO Code	Unique country identifier (to match data at country level)
Country Name	Written name of each country
Keywords 1 & 2	Up to three columns of policy keywords to quickly find relevant policies
Policy Type	Initial categorization of policies that can easily be adapted
Scale	Policy scale: sub-national, national, super-national
First Year	When a policy was first introduced
Year 1,2,3	Up to three important changes of each recorded policy
Descriptors	Policy description with more detail than the keyword variables
Title	A descriptive title for each policy
Link	Online link whenever available
Related File	PDF or other text document whenever available
Abstract	Short policy summary whenever available

Supplementary Table 1. Main Dataset Variable Overview. The main database itself has 15 variables as listed here.

Variable	Explanation	Source
ISO3	Three digits ISO country identifier	[60]
FIPS	Two digits FIPS country identifier	[60]
ISO2	Two digits ISO country identifier	[60]
name	Full country name	[60]
area	Country area in Km^2	[61]
pop2005	Country population ca. 2005	[61]
region	World region identifier (6 continents)	[62]
subregion	Sub-region identifier (23 regions)	[62]
lon	Country longitude	[63]
lat	Country longitude	[63]
number_policies	Number of agri-environmental policies	new
number_policies_eu	Same as above but including EU policies	new
bayesian_corruption	Bayesian Corruption Index	[20]
green_seats_parl	Seats of green party in parliament	[64]
votes_green	Votes for green party in election	[64]
epi_agr	Environmental Performance Index: Agriculture	[47]
environmental_performance_index	Environmental Performance Index: All Domains	[47]
security_property	Security of Private Property Index	[65]
env_expenditure	Government's environmental expenditure share	
sus_en_pol	Sustainable Environmental Policies: All	[66]
sus_en_pol_env	Sustainable Environmental Policies: Environment	[66]
sus_en_pol_gl_prot	Sustainable Environmental Policies: Global Protect.	[66]
property_rights_protection	Property Rights Protection index	[67]
undp_hdi	Human Development Index of the UN	[68]
wdi_gdpcapcon2010	GDP per capita in constant 2010 US$	[61]
wdi_gdpgr	GDP share of the agricultural sector	[61]
wdi_gdppppcur	GDP in purchasing power parity, current US$	[61]
pol_inst_env	Quality of Political Institutions: Environment	[66]
rule_based_gov	Rule Based Governance Indicator	[61]
epi2018score	2018 Environmental Performance Score	[47]
stringency	Perceived Environmental Policy Stringency	[19]
enforcement	Perceived Environmental Policy Enforcement	[19]

Supplementary Table 2. Variables in the Country-Level Dataset. This dataset is aggregated over all countries and years and contains the number of policies per country and several other characteristics.

Variable	Explanation	Source
id	unique grid-cell identifier	[27]
x	longitude of the centroid of each grid-cell	[27]
y	latitude of the centroid of each grid-cell	[27]
border	unique country-pair identifier	[27]
log_erosion	natural log of the rate of soil erosion	[27]
more_soil_policies	indicator variable which country of each country-pair (border) has implemented more soil policies	new
border distance	Distance from the centroid of each grid-cell to the relevant national border	[27]
rainfall erosivity	the R factor from the RUSLE of a grid-cell	[27]
soil erodibility	the K factor from the RUSLE of a grid-cell	[27]
lengths and angles of slopes	The LS factor from the RUSLE of a grid-cell	[27]
environmental_performance_index	Table 2	
wdi_gdpcapcon2010	Table 2	
property_rights_protection	Table 2	
bayesian_corruption	Table 2	

Supplementary Table 3. Variables for the Soil Erosion Analysis. The soil erosion analysis is based on 15,687,325 grid-cells covered with cropland that is located within 80 Km to a national border. This table shows the used variables.
